# On the Use of Entropy to Improve Model Selection Criteria

**DOI:** 10.3390/e21040394

**Published:** 2019-04-12

**Authors:** Andrea Murari, Emmanuele Peluso, Francesco Cianfrani, Pasquale Gaudio, Michele Lungaroni

**Affiliations:** 1 Consorzio RFX (CNR, ENEA, INFN, Universita’ di Padova, Acciaierie Venete SpA), 35127 Padova, Italy; 2 Department of Industrial Engineering, University of Rome “Tor Vergata”, 00133 Roma, Italy

**Keywords:** Model Selection Criteria, Bayesian Information Criterion (BIC), Akaike Information Criterion (AIC), Shannon Entropy, Geodesic Distance

## Abstract

The most widely used forms of model selection criteria, the Bayesian Information Criterion (BIC) and the Akaike Information Criterion (AIC), are expressed in terms of synthetic indicators of the residual distribution: the variance and the mean-squared error of the residuals respectively. In many applications in science, the noise affecting the data can be expected to have a Gaussian distribution. Therefore, at the same level of variance and mean-squared error, models, whose residuals are more uniformly distributed, should be favoured. The degree of uniformity of the residuals can be quantified by the Shannon entropy. Including the Shannon entropy in the BIC and AIC expressions improves significantly these criteria. The better performances have been demonstrated empirically with a series of simulations for various classes of functions and for different levels and statistics of the noise. In presence of outliers, a better treatment of the errors, using the Geodesic Distance, has proved essential.

## 1. Bayesian and Information Theoretic Model Selection Criteria

Assessing the quality of hypotheses is an essential step in science. The so called scientific method indeed consists of a continuous iterative process of hypothesis formulation, testing with experiments or observations and refinement [1,2]. To subject hypotheses to quantitative analysis, they have to be expressed as mathematical models. Consequently, the identification and selection of models is a fundamental task scientists are confronted with. Of course, no model can perfectly represent the real world and therefore the goal typically consists of selecting the best model in a set of candidates. To this end, the statistical approach to model selection is relatively recent. This methodology is based on the evaluation of suitable criteria, which qualify the models by striking a balance between reproducing the data and avoiding overfitting. The two most widely used model selection families of indicators are the Bayesian Information Criterion (BIC) [3] and the Akaike Information Criterion (AIC) [4]. The BIC criterion is derived in the framework of Bayesian theory and it is meant to maximize the posterior probability of a model given the data. AIC is based on the Kullback-Leibler Divergence [5] and essentially estimates the information lost by a given model. Therefore it is assumed that the less information a model loses, the higher its quality. 

The theoretical derivations of these metrics result in the following unbiased forms of the criteria:

(1) BIC=−2ln(L)+kln(n)

(2) AIC=−2ln(L)+2k

where L is the likelihood of the model given the data, k the number of estimated parameters in the model and n the number of entries in the database. Both BIC and AIC are metrics to be minimized; they favour models with a high likelihood but implement a penalty for complexity (the term proportional to k). 

In most practical applications, the likelihood of a model is not easy to calculate. In the exact sciences, for example, many models are expressed as deterministic equations, deductively obtained from previous theories and often dependent on the approximations made for their derivation. The probabilities of the assumptions, introduced to develop the competing models, are normally unknown and the likelihood of the model estimates is not computable. Even in the case of statistical models, the data can be insufficient or affected by excessive noise, the number of parameters can be excessive etc., rendering the computation of the likelihood virtually impossible. To overcome the practical difficulties of reliably calculating the likelihood, the typical solution consists of assuming that the model and data errors are identically distributed and independently sampled from a normal distribution. If this hypothesis is valid, it can be demonstrated that the BIC can be written (up to an additive constant, which depends only on the number of entries in the database and not on the model):

(3) $$BIC=n\cdot \ln\left(\sigma_{\left(\epsilon \right)}{}^{2}\right)+\;k\cdot \ln\left(n\right)$$

where $\sigma_{\left(\epsilon \right)}{}^{2}$ is the variance of the residuals.

Similar assumptions allow expressing the AIC criterion as:

(4) AIC=n⋅ln(MSE)+ 2k

where MSE is the mean-squared error of the residuals.

Equations (3) and (4), whose derivation is detailed in [5], are by far the most widely used forms of BIC and AIC, in which the error variance and the MSE are calculated on the basis of the residuals, the differences between the data and the estimates of the models.

As can be easily appreciated by inspection of Equations (3) and (4), in the practical versions of BIC and AIC, the statistical information originally in the likelihood is now reduced to the variance and MSE of the residuals. It is therefore legitimate to ask whether some additional statistical information about the distribution of the residuals can be included in the criteria to improve their performance. A good indicator of the residual distribution is the Shannon entropy, which can be profitably used to improve both AIC and BIC. The following sections are meant to support this heuristic empirically with a systematic series of numerical tests. 

The paper is structured as follows. The rationale, behind the use of the Shannon entropy of the residuals, to improve Bayesian and information theoretic model selection criteria, is provided in Section 2. Extensive numerical tests, showing the advantages of the new proposed versions of the AIC and BIC criteria, are the subject of Section 3. The issues posed by noise of different statistics and outliers and a more robust treatment of the measurement errors are discussed in Section 4. Summary and future developments are the subject of the last Section 5.

## 2. Rationale for the Use of Entropy in Model Selection Criteria 

The motivation behind the use of the Shannon entropy in selection criteria is based on the observation that, if a model were perfect, the residuals should be due only to the noise affecting the data. Assuming additive random noise, models, whose residuals present a more uniform probability density function (pdf), should therefore be preferred. Indeed the residuals of inferior models are expected to present patterns that reflect the trends in the data not properly identified by the models. Of course, the need to favour models, with a more uniform distribution of the residuals, can be quantified using the Shannon entropy, which assumes its maximum value exactly in the case of data of uniform probability. These considerations suggest testing the performance of the following modified versions of the BIC and AIC criteria:

(5) $$BIC_{H}=n\cdot \ln\left(\frac{\sigma_{\left(\epsilon \right)}{}^{2}}{H}\right)+\;k\cdot \ln\left(n\right)$$

(6) $$AIC_{H}=n\cdot \ln\left(\frac{MSE}{H}\right)+\;2k$$

where H indicates the Shannon entropy of the residuals: H = −$\underset{i}{\sum }p_{i}\;lnp_{i}$ and *p_i_* is the probability of the i-sm residual. The working hypothesis investigated in this paper is whether including the entropy of the residuals in the BIC and AIC can improve their discrimination capability. The underlying heuristics is that, other things being equal, models with a more uniform distribution of the residuals, and therefore with a higher H, are to be considered preferred solutions. Indeed it should be remembered that the BIC and AIC criteria are metric to be minimised. 

The main justification of the empirical considerations summarised in the previous paragraph is that, in many scientific applications, particularly in physics and chemistry, the data available are measurements obtained with complex experimental devices, practically always affected by some form of noise. The sources of noise are many, additive and independent and one can therefore invoke the Central Limit Theorem, considering these sources as random variables. It is therefore fully reasonable to assume that the probability distribution of the noise is Gaussian. The strategy of favouring models with higher H seems therefore justified, since it is also in harmony with the assumptions leading to the expression of the criteria given in Equations (3) and (4). 

A more formal justification of the expressions (5) and (6) requires the demonstration that the new forms of the indicators are asymptotically unbiased (their faster convergence will be shown numerically in the next sections). This can be seen under the assumptions that that the residuals are normally distributed with vanishing expectation value and homoscedastic (constant variance σ). The just mentioned hypotheses are exactly the ones used to derive the practical versions of the BIC and AIC, relations (3) and (4). Under these assumptions, the Shannon entropy reads

(7) $$H=\sum_{i=1}^{n}p_{i}\left(-\ln p_{i}\;\right)=\sum_{i=1}^{n}\frac{1}{\sqrt{2\pi \;}\sigma \;}e^{-\frac{{\left(y_{i}-\;{\hat{y}}_{i}\right)}^{2}}{2\sigma^{2}}}\;\left[\frac{{\left(y_{i}-\;{\hat{y}}_{i}\right)}^{2}}{2\sigma^{2}}+\ln\left(\sqrt{2\pi \;}\sigma \right)\right]$$

where $y_{i}$ denote the measured values and ${\hat{y}}_{i}$ are the predictions, which depend on the adopted models. In the limit n→∞ the summation can be replaced by the integral across the entire range of the probability distribution. The Shannon entropy can be then explicitly computed finding:

$$H=\;\frac{1}{2}+\ln\left(\sqrt{2\pi \;}\sigma \right).$$

The above expression does not contain the predictions $\;{\hat{y}}_{i}$ and thus it does not depend on the chosen model. Therefore, the Shannon entropy asymptotically provides the same contribution for all models, implying that the new BIC_H_ and AIC_H_ criteria coincide with the standard ones in the limit n→∞.

It should be also mentioned that favouring models with higher entropy of the residuals can be advisable even when these assumptions are not completely satisfied, for example in case of outliers. This matter will be discussed in more detail in Section 4. It should also be mentioned that, in order to compare coherent values, the entropy of the various models has been calculated after normalising the residuals in the interval (−1, +1).

## 3. Numerical Tests for Random Gaussian Noise

To test the proposed approach, and in particular the performance of BIC_H_ and AIC_H_ (Equations (5) and (6)), a series of systematic numerical tests has been performed. Synthetic data have been generated for four main families of models: polynomials, power law monomials, exponentials and power law monomials multiplied by squashing terms. Therefore, the basic functions, typically used in building deterministic models for the exact sciences, have been considered [6]. Consequently, for coherence sake, the notation is typical of physics and chemistry; y indicates the dependent variables and x the generic regressor, which can be thought of as time or any other suitable quantity. The number of parameters of each model is indicated with *k* and includes the exponents and the multiplicative constants of the independent variables and their functions. As examples, in the accounting used in the paper, the number of parameters of a simple exponential *a exp(bx^c^)* is 3, as for a sine function *a sin(bx^c^).* In the case of power laws and polynomials, of course, the number of parameters is the sum of the coefficients and the exponents of the various terms. 

The results have been very positive and the proposed new versions of the criteria have always outperformed the traditional ones. To substantiate these statements, some examples are reported in this section. All the plots and results shown in the rest of the paper refer to individual data sets (individual realisations of the noise). This choice has been motivated by the consideration that this is the most relevant situation in practice, since typically only single sets of measurements are available. In any case, the performances reported are absolutely representative of the investigated situations, as verified with tens of different realisations of the noise.

**Figure 1:**
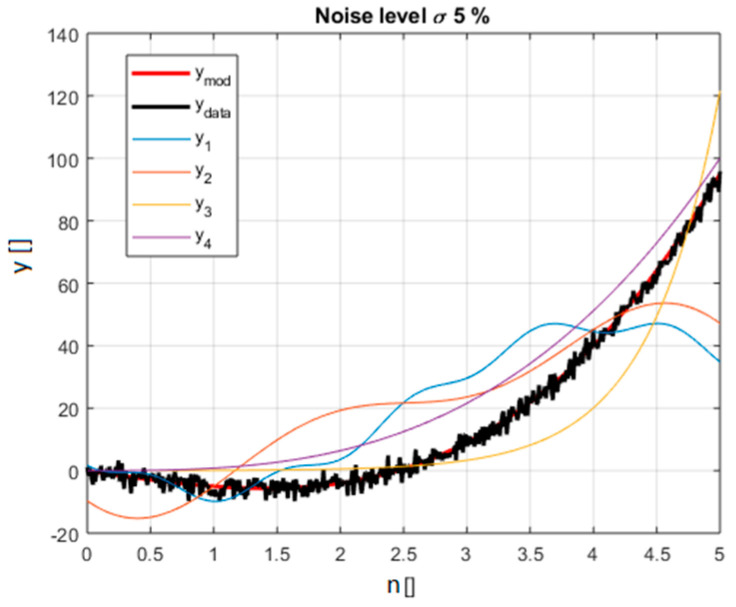
The various lines depict the polynomial functions considered in the selection and reported in Table 1; the different functions are identified by a corresponding colour explained in the legend. The synthetic data including noise is in black (for noise level 5% of the average value of the right model in red).

The models in the polynomial family, covered as a representative example, are reported in Table 1. The equation used to generate the synthetic data is called ref: $x^{3}-6x$. The choice of the alternative models, and the values of their numerical parameters, has been driven by the intention to obtain functions, which can closely fit the data. In this way, the indicators are faced with a difficult task because they have to discriminate between closely competing models. Gaussian noise has been added to the points obtained with the reference equation. The standard deviation of the noise has been scanned in an interval ranging from 5% to 25% of the average value of the ref function in the interval considered. A graphical view of the various models and the synthetic data is shown in Figure 1. A systematic comparison of the difference between the BIC and AIC indicators for all the models of Table 1 has been performed. 

**Figure 2:**
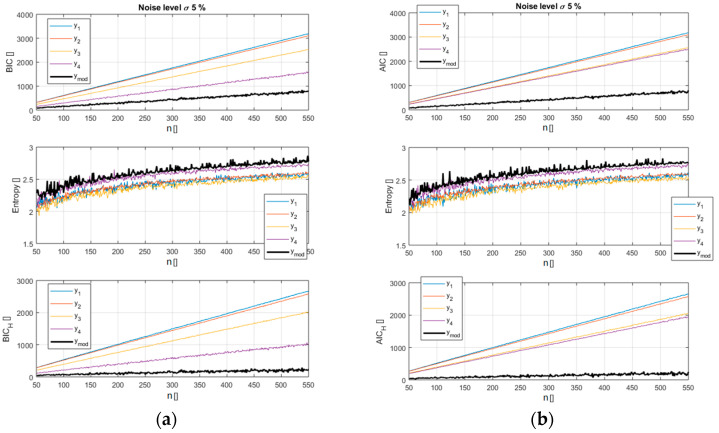
Top plots: values of the traditional BIC (**a**) and AIC (**b**) for the models of Table 1 versus the number of entries in the database. Middle plots: entropy H for the models of Table 1. Bottom plots: values of the new BIC_H_ and AIC_H_ for the models of Table 1 versus the number of entries in the database.

**Figure 3:**
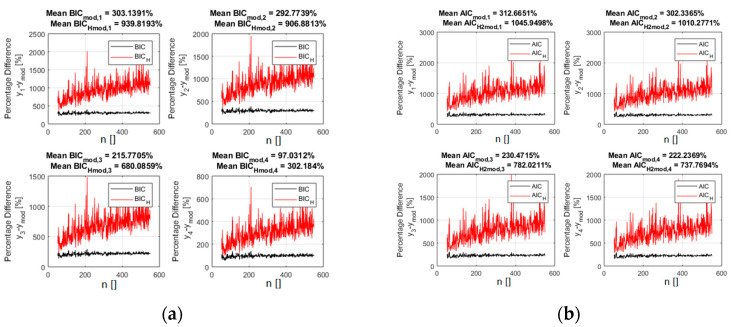
The percentage difference between the values of the indicators for the right polynomial model and all the competing ones. BIC_H_ (**a**) and AIC_H_ (**b**) are in red and the traditional form of the BIC (**a**) and AIC (**b**) are reported in black. The x axis shows the number of entries in the database.

The analysis reveals how the new versions of the indicators, BIC_H_ and AIC_H_, provide a better separation between the models and allow an easier identification of the right function. A pictorial view of this information is reported in Figure 2 and Figure 3 for a scan in the number of data available (from 50 to 550). It should be noted that in these figures, as in all the remaining ones in the paper, the x axis indicates the number of entries used for the calculations. So the value of the BIC_H_ and AIC_H_, reported in correspondence to the abscissa x = 100 for example, has been calculated using 100 synthetically generated data points. The case reported in Figure 2 and Figure 3 refers to a noise of 5% of the average value of the reference function (the exact case shown in Figure 1). Figure 2 depicts how the entropy of the correct model residuals is higher than that of the other functions; it is therefore only natural that including this parameter in the selection criteria should improve the separation between the right model and the wrong candidates.

Figure 3 indeed reports the difference in percentage between the indicator values for the alternative models and the correct one. Including the entropy in the calculations of the indicators, according to relations (5) and (6), improves the separation between the right models and the incorrect candidates. It is important to notice that this separation keeps increasing with the number of points, indicating good asymptotic properties. A scan in the noise, whose results are reported in supplementary materials, reveals that the new indicators continue also to provide better separation between the optimal and the incorrect models for all the levels of noise investigated. 

**Figure 4:**
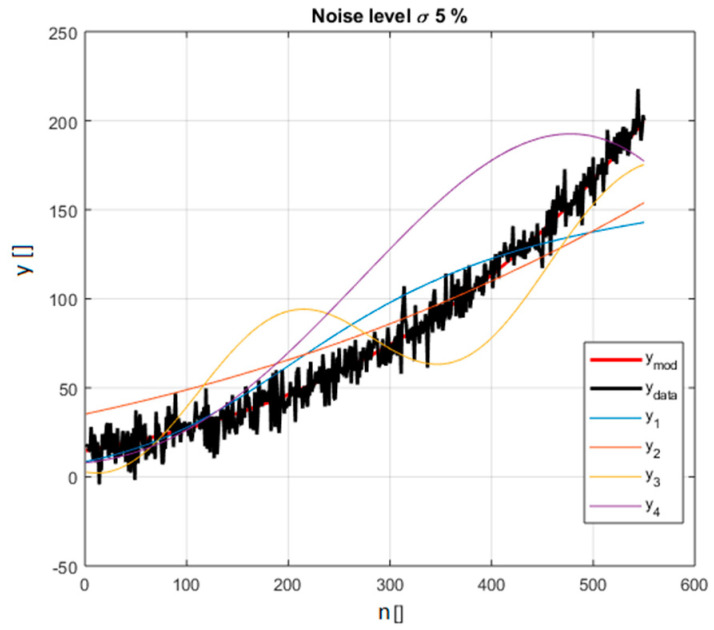
The power law monomials considered in the selection and reported in Table 2; the synthetic data including noise is in black (for noise level 5% of the average value of the right model in red).

As an additional example, a set of functions belonging to the class of power law monomials is shown next. This is another family of equations, which have the form $y=const\;x_{1}^{e1}\;\;x_{2}^{-e2}\ldots .\;x_{n}^{en}$ and are often encountered in practice. Indeed power law monomials are among the most popular types of functions, particularly in the investigations of scaling laws, to quantify how the properties of systems change with dimensions. The capability of properly identifying power law monomials is therefore particularly important both in theory and practice. The reference equation, used to generate the synthetic data and therefore to be considered the right model, is $y=2.5\;x_{1}^{2.5}\;\;x_{2}^{-0.75}\;\;x_{3}^{2.5}\;\;\;\forall \;x_{1}\in \left[1,\;2\right],\;x_{2}\in \left[20,30\right],\;\;x_{3}\in \left[5,\;8\right]$. The other competitive models are reported in Table 2 and Figure 4. 

**Figure 5:**
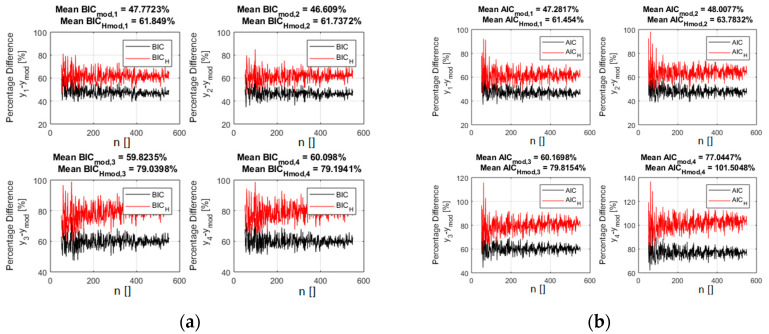
The percentage difference between the values of the indicators for the right power law monomial model and all the competing ones. BIC_H_ (**a**) and AIC_H_ (**b**) are in red and the traditional form of the BIC (**a**) and AIC (**b**) are reported in black.

Again the standard deviation of the noise has been scanned in an interval 5% to 25% of the average value of the ref function in the interval considered. A scan in the number of data available covers also the realistic range of 50 to 550 entries. The results of a comparison between the BIC and AIC indicators are provided in detail in supplementary materials, which reports in particular a scan in the level of noise again expressed as the percentage of the average value of the reference function. In Figure 5, the values of BIC_H_ and AIC_H_ are shown for the case of 5% noise (the specific example of Figure 4).

Again, the inclusion of the entropy of the residuals, according to Equations (5) and (6), improves the discriminatory capability of the indicators practically for all the levels of noise investigated and for all the number of entries in the database. Qualitatively the same results have been obtained also for the other families of functions investigated such as the exponentials, whose general mathematical form is f(x) = ab^cx+d^. In all cases simulated, the BIC_H_ and AIC_H_ versions of the indicators have always shown a better capability of discriminating the right models among a number of very similar candidates. The trends reported for the polynomials and power law functions are confirmed; the improvements tend to increase with the number of entries until saturation and they remain significant even for quite high levels of noise. Even better results have been obtained for the other more complex families of functions investigated such as power laws multiplied by squashing terms. Functions such as the sigmoid, the hyperbolic tangent etc. are called squashing functions because they compress the input into a small interval: the range of [−1, 1] for the sigmoid y = e^x^/(e^x^ +1). In the case of these more complex functions, it has been found that the BIC_H_ and AIC_H_ versions of the indicators not only have better discriminating capabilities but also, in several cases, allow identifying the right model when the traditional indicators fail. An example is reported in Table 3. 

The functions of Table 3 are sufficiently complex, and the other candidate models of enough quality, that the traditional versions of the BIC and AIC have problems detecting the model generating the data. The new indicators, BIC_H_ and AIC_H_, fare much better for several combinations of noise level and number of entries. In particular, they systematic manage to identify the right model for relative low numbers of inputs, as reported in Table 4 for a couple of cases.

It can therefore be concluded that, taking into account the entropy of the residuals improves the quality of the indicators, thanks to the additional statistical information provided. The case of data affected by noise of different statistics and outliers, and a more advanced treatment of the errors, are discussed in the next section.

## 4. Additional Topics: Noise of Different Statistics, Outliers and Geodesic Distance

**Figure 6:**
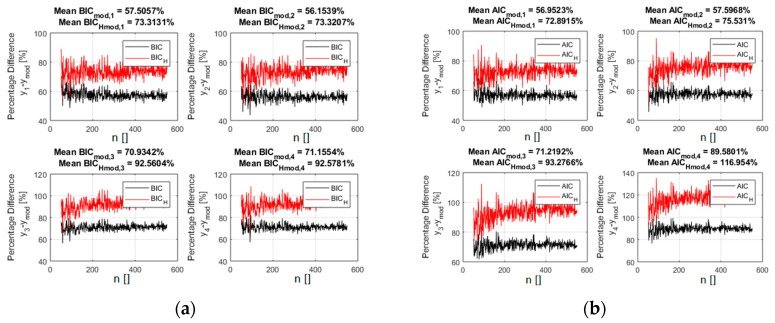
The percentage difference between the values of the indicators for the right power law monomial and all the competing ones (Table 2). The added noise in this case presents a uniform distribution of standard deviation equal to 5% of the average value of the right model. BIC_H_ (**a**) and AIC_H_ (**b**) are in red and the traditional form of the BIC (**a**) and AIC (**b**) are reported in black.

Given the encouraging results obtained with Gaussian noise reported in the previous section, additional tests have been performed to investigate the generality of the new versions of the indicators BIC_H_ and AIC_H_. First, a systematic analysis of noise of different statistics has been studied. One particularly relevant case is uniform noise, since some experimental measurements in the sciences can present uncertainties, which can be approximated by such a distribution. As expected also from intuitive considerations, the advantages of adopting the BIC_H_ and AIC_H_ versions of the model selection criteria are even larger for this case. Indeed, for additive uniform noise statistics, the residuals of the right model should be even more uniform, and therefore maximize even more the Shannon entropy, than when the data are affected by Gaussian noise. To illustrate this point, Figure 6 shows the improvement in the values of the indicators for the case of power law monomials with 5% of added noise (the same models reported in Table 2). A comparison between Figure 5 and Figure 6 indeed confirms the even better performance of BIC_H_ and AIC_H_ in the case of uniform noise. 

The proposed new formulation of the indicators has also been applied to databases affected by outliers. It has to be said that, in this case the improvements provided by BIC_H_ and AIC_H_ are still noticeable but marginal. To improve this not completely satisfactory situation, it has been decided to implement a better treatment of the errors. As mentioned, even BIC_H_ and AIC_H_ measure the quality of the fit typically with a quantity proportional to the sum-of-squares of the distances between the data and the model predictions (the variance and the MSE respectively). In this way, they are implicitly adopting the Euclidean distance to calculate the (dis)similarity between data points and predictions. However the Euclidean distance implicitly requires considering all data as single infinitely precise values. On the other hand, the derivations of Equations (3) and (4) are predicated on the data presenting a Gaussian distribution. It would be therefore more consistent to consider the measurements not as points, but as Gaussian distributions when calculating the residuals. Modelling measurements not as punctual values, but as Gaussian distributions, requires defining a distance between Gaussians. The most appropriate definition of distance between Gaussian distributions is the geodesic distance (GD), on the probabilistic manifold containing the data, which can be calculated using the Fischer-Rao metric [7,8]. For two univariate Gaussian distributions $p_{1}\left(x|\mu_{1},\sigma_{1}\right)$ and $p_{2}\left(x|\mu_{2},\sigma_{2}\right)$, parameterised by their means $\mu_{i}$ and standard deviations $\sigma_{i}$, the geodesic distance GD is given by:

(8) GD(p1||p2)=2ln1+δ1−δ=2tanh−1δ,  where δ=[(μ1−μ2)2+2(σ1−σ2)2(μ1−μ2)2+2(σ1+σ2)2]12

**Figure 7:**
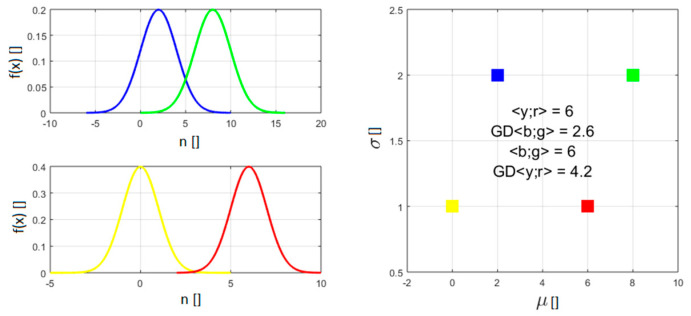
Examples to illustrate how the GD determines the distance between two Gaussians. The two couples of pdf in the figure have the same difference in mean but different σ. The geodesic distance between the two with higher σ is much smaller. In the right plot GD indicates the geodesic distance and <> the Euclidean distance.

The meaning of GD can be appreciated by inspecting Figure 7, which reports the distance between two couples of Gaussian distributions. The distance between the means of the members of the two couples is the same. On the other hand, the Gaussian pdfs of one couple have a standard deviation an order of magnitude higher the other. The distance between the pdfs with higher standard deviation is therefore significantly lower than the one of the more concentrated pdfs, which is intuitively and conceptually correct since they overlap much more.

**Figure 8:**
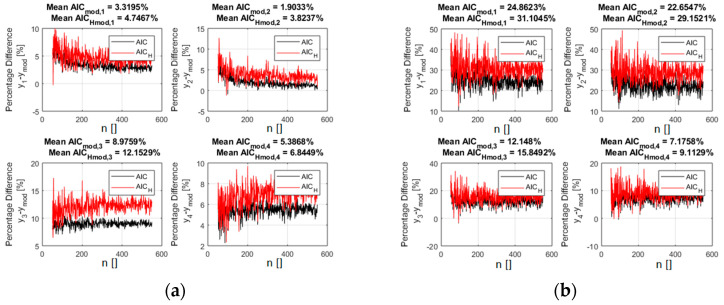
The percentage difference between the values of the AIC indicator for the right polynomial function and all the competing ones (reported in Table 1). Left plot (**a**): residuals calculated with the GD. Right plot (**b**): residuals calculated with the Euclidean distance. The added noise in this case presents a distribution obtained with relation 8. AIC_H_ is in red and the traditional form of the AIC is reported in black. The fraction of outliers is 15%.

As mentioned, the implementation of the Geodesic distance has proved particularly useful to handle outliers. To investigate this aspect, the noise, added to the data of the generating functions, consists of two Gaussians: one of zero mean and standard deviation $\sigma_{I}$, which simulates additive noise, and one of non zero mean calculated with the following formula:

(9) $$\mu_{2}=2\left(\sigma_{I}+\sigma_{II}\right)/100\cdot \bar{f\left(x\right)}$$

where $\sigma_{II}$ is the standard deviation of the second Gaussian, meant to represent the outliers and $\bar{f\left(x\right)}$ is the average of the generating function in the considered interval. With this type of “noise”, including outliers, calculating the residuals with the GD has always improved the performance of the indicators. In many cases, it has even been possible to properly identify the right model even when it was prohibitively difficult with the traditional Euclidean distance. A representative example is shown in Figure 8, where the AIC_H_ indicator is reported for the case of the polynomial functions reported in Table 1.

The outliers are 15% of the 400 inputs points. The standard deviation of the two Gaussians is: $\sigma_{I}=10\%$ and $\sigma_{II}=30\%$, where as usual the percentages refer to the average value of the correct function in the interval considered. The use of the GD allows a clear identification of the right model, whereas with the Euclidean distance, the incorrect candidates have very similar values of both indicators. It should be mentioned that the BIC indicator provides results very similar to the AIC. Moreover, if the added random noise is more uniform, the separation between the right and the wrong models becomes even higher, even in presence of outliers.

## 5. Conclusions

In the most widely used versions of the model selection criteria BIC and AIC, the statistical information about the residuals is limited to the MSE and variance, because often it is very difficult, if not impossible, to actually compute the likelihood. Therefore additional information about the distribution of the residuals would be useful. Taking into account the Shannon entropy, to favour models with a more uniform distribution of the residuals, has proven to be very advantageous in all the numerical cases investigated. The new form of the model selection criteria, BIC_H_ and AIC_H_, has always allowed a better separation between the right model and the incorrect competitors. These results have been obtained for different classes of functions, various levels of noise and scanning the number of entries in the databases. The proposed version of the statistical indicators outperforms the old one also when the data are affected by noise of different statistics. The implementation of the Geodesic Distance has proved essential to counteract the negative impact of outliers. On the other hand, some cautionary words are in place. First, it should be remembered that the proposed criteria are based on simple heuristic considerations and have been tested only empirically; the numerical simulations have been extensive and conclusive but certainly the improved performance are to be considered proved exclusively for the classes of functions and the typologies of the noise investigated so far. Additional work is required to devise theoretical justifications for BIC_H_ and AIC_H_. Moreover, even for the cases when the performances of the new indicators outperform the classic versions of BIC and AIC, it is recommended that their results are complemented with other forms of statistical inference for model checking, based for example on Bayesian techniques. 

With regard to future lines of investigation, in terms of applications, BIC_H_ and AIC_H_ can be used in various disciplines not only for model selection but also for exploration of the operational space using genetic algorithms. One case is point is Magnetic Confinement Nuclear Fusion [9,10], in which the model versions of AIC and BIC are already extensively implemented [11,12,13,14]. One application, which can certainly profit form the proposed new version of the indicators, is the identification of the most appropriate solution of difficult inverse problems such as the reconstruction of the magnetic fields and the tomographies [15,16,17,18,19]. 

From a methodological point of view, it should be considered whether other information to complement the entropy can be profitably taken into account. Indeed, additional knowledge about the likelihood might be available that, even if insufficient to calculate it reliably, might be used to obtain additional statistical constraints on the residuals. Also other definitions of the entropy could be investigated. It should also be mentioned that, when possible, the performance of the proposed indicators could be compared with complementary Bayesian model selection approaches [20,21].

## Figures and Tables

**Table 1:** Example of selection for data generated with a polynomial model. The model used to generate the data is the reference one. The number of parameters of the models is indicated with k.

#	Model	k
1	20−27sin(x+0.61)−2.86 cos(6.13x)	8
2	20+12.8 cos(2x−16)−27 cos(x−0.87)	9
3	$1.5\cdot 10^{-2}\;\exp\left(1.8x\right)$	3
4	$0.8\;x^{3}$	2
ref	$x^{3}-6x$	4

**Table 2:** Example of selection for data generated with a model in the form of a power law monomial. The model used to generate the data is the reference one. The number of parameters of the models is indicated with k.

#	Model	k
1	$\frac{0.204x_{2}}{\sin\left(x_{1}\left(\frac{0.46}{x_{1}^{8.72}}+\frac{0.61}{x_{2}}\right)\right)}$	8
2	$0.258\left(x_{3}^{3.08}-x_{3}\right)-0.03\sin\left(x_{3}^{-12.62}\right)$	6
3	$31.23\left(x_{1}^{2.21}-\sin\left(x_{2}\right)\right)$	4
4	$50+10.45x_{1}x_{3}\sin\left(1.07x_{3}\right)$	6
ref	$2.5\;x_{1}^{2.5}\;\;x_{2}^{-0.75}\;\;x_{3}^{2.5}$	4

**Table 3:** Example of selection for data generated with a model in the form of a power law monomial multiplied by a squashing term. The model used to generate the data is the reference one. The number of parameters of the models is indicated with k.

#	Model	k
1	$y_{1}=\;1.68\cdot 10^{4}\sin\left(\frac{x_{1}}{x_{2}^{4.18}}\right)$	2
2	$y_{2}=3\;x_{2}\exp\left(-x_{3}^{9.48}\right)$	2
3	$y_{3}=17.87\;{\left(\frac{x_{1}}{x_{2}^{0.45}}\right)}^{0.47}$	3
4	$y_{4}=3.5\;x_{1}^{0.4}\;\;x_{2}^{0.8}$	3
ref	$y_{ref}=2\;x_{1}^{0.6}\;\;x_{2}^{1.1}\;\frac{1}{1+exp\left(-2\;x_{3}^{1.5}\right)}\;\;\;\;\;\;\;$	4

**Table 4:** Comparison of the classification obtain with BIC, AIC and BIC_H_, AIC_H_ for the models reported in Table 3. The model denoted as ref is the one used to generate the synthetic data.

**Noise Level 20%—Points Number 300**				**Noise Level 30%—Points Number 300**			
**Model Rank**	**AIC**	**Model Rank**	**AIC_H_**	**Model Rank**	**AIC**	**Model Rank**	**AIC_H_**
$\;y_{1}$	805.3	$y_{ref}$	460.4	$y_{3}$	1129.7	$y_{ref}$	776.9
$y_{ref}$	807.4	$y_{1}$	461.4	$y_{ref}$	1136.9	$y_{3}$	779.4
$y_{2}$	837.9	$y_{2}$	484.1	$y_{2}$	1141.6	$y_{2}$	797.8
$y_{3}$	923.1	$y_{3}$	579.9	$y_{1}$	1181.0	$y_{1}$	837.4
$y_{4}$	1136.6	$y_{4}$	794.8	$y_{4}$	1280.5	$y_{4}$	943.3
**Noise Level 20%—Points Number 500**				**Noise Level 30%—Points Number 500**			
**Model Rank**	**BIC**	**Model Rank**	**BIC_H_**	**Model Rank**	**BIC**	**Model Rank**	**BIC_H_**
$y_{1}$	1262.8	$y_{ref}$	693.5	$y_{1}$	1720.1	$y_{ref}$	1160.9
$y_{ref}$	1263.1	$y_{1}$	703.2	$y_{ref}$	1720.5	$y_{1}$	1161.9
$y_{2}$	1310.0	$y_{2}$	748.3	$y_{3}$	1741.1	$y_{3}$	1180.1
$y_{3}$	1432.5	$y_{3}$	872.6	$y_{2}$	1761.5	$y_{2}$	1188.4
$y_{4}$	1582.2	$y_{4}$	1025.6	$y_{4}$	1965.4	$y_{4}$	1404.4

## Supplementary Materials

The following are available online at https://www.mdpi.com/1099-4300/21/4/394/s1.
