# Supplementary material for: On the Use of Entropy to Improve Model Selection Criteria
Source: Entropy (Basel). 2019 Apr 12;21(4):394. doi: 10.3390/e21040394 (PMC7514877; doi:10.3390/e21040394)

entropy

MDPI

On the Use of Entropy to Improve Model Selection Criteria

Andrea Murari [1], Emmanuele Peluso [2], Francesco Cianfrani [2], Pasquale Gaudio [2] and Michele Lungaroni [2,*]

[1] Consorzio RFX (CNR, ENEA, INFN, Universita' di Padova, Acciaierie Venete SpA), 35127 Padova, Italy; andrea.murari@euro-fusion.org

[2] Department of Industrial Engineering, University of Rome "Tor Vergata", 00133 Roma, Italy; emmanuele.peluso@uniroma2.it (E.P.); francesco.cianfrani@uniroma2.it (F.C.); gaudio@ing.uniroma2.it; (P.G.)

* Correspondence: michele.lungaroni@uniroma2.it; Tel.: +39 0672597196

Received: 21 January 2019; Accepted: 10 April 2019; Published: date

1. Scan in the noise for the models belonging to the polynomial class.

<table>
<tr><th colspan="2">Gaussian Noise 10% - AIC (Figures S1–S3)</th></tr>
<tr><td rowspan="2">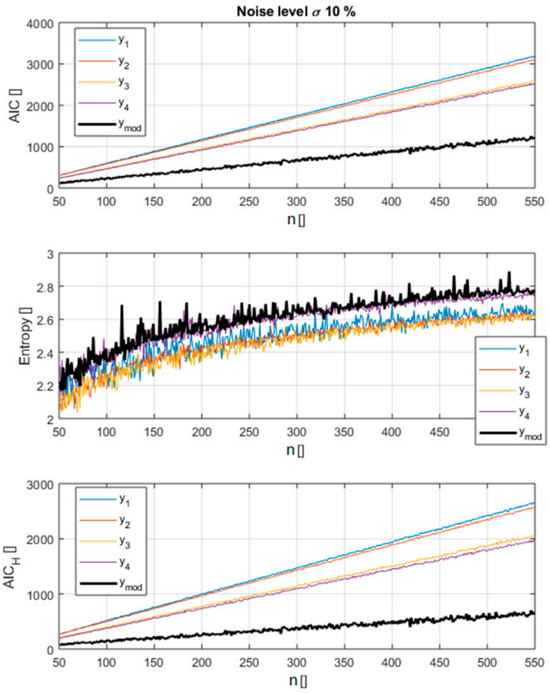
</td>
<td>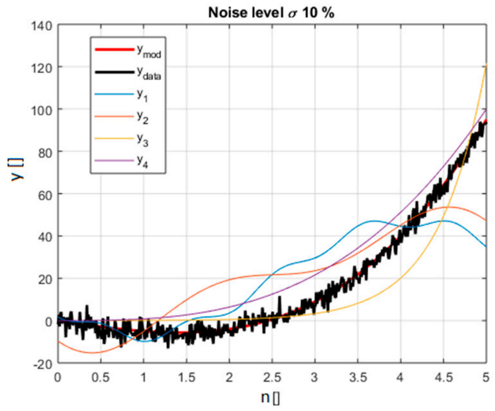
</td></tr>
<tr><td>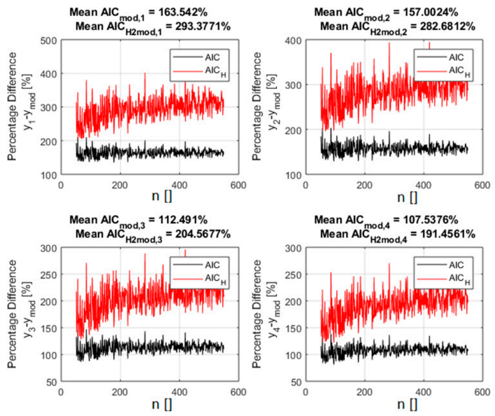
</td></tr>
<tr><th colspan="2">Gaussian Noise 20% - AIC (Figures S4–S6)</th></tr>
<tr><td colspan="2"></td></tr>
</table>

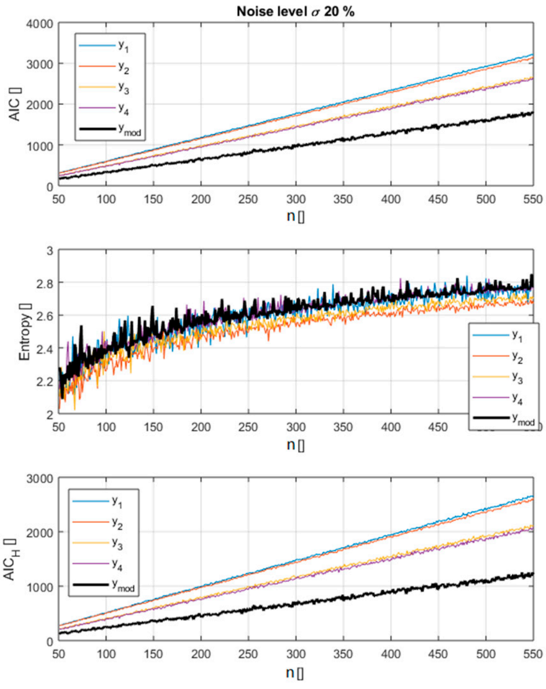

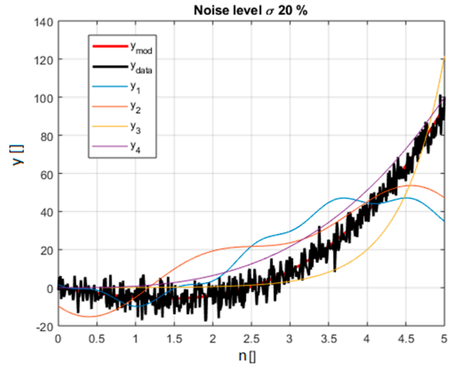

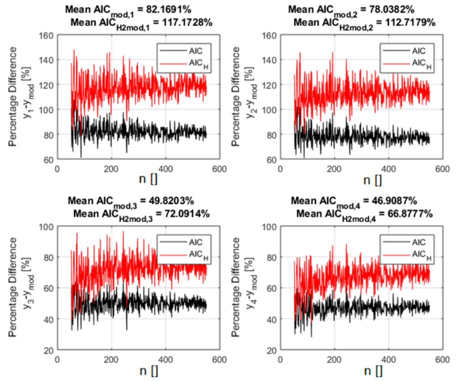

Gaussian Noise 25% - AIC (Figures S7–S9)

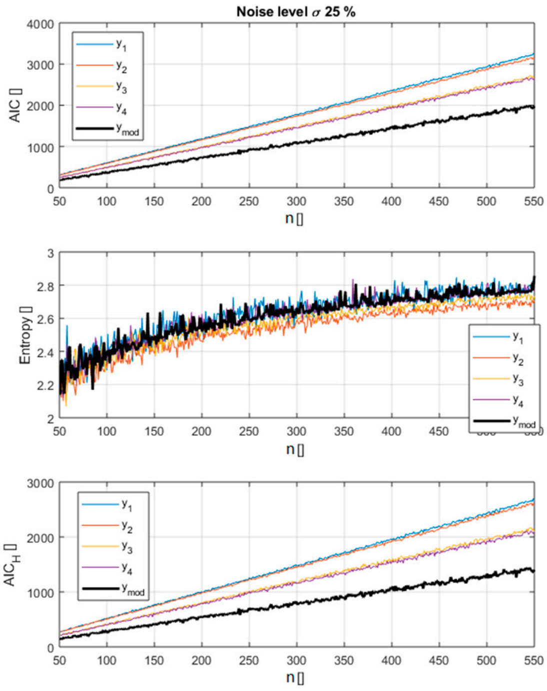

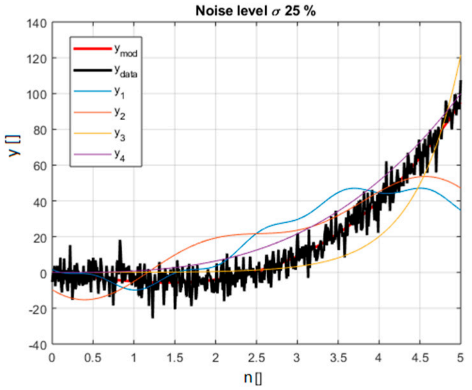

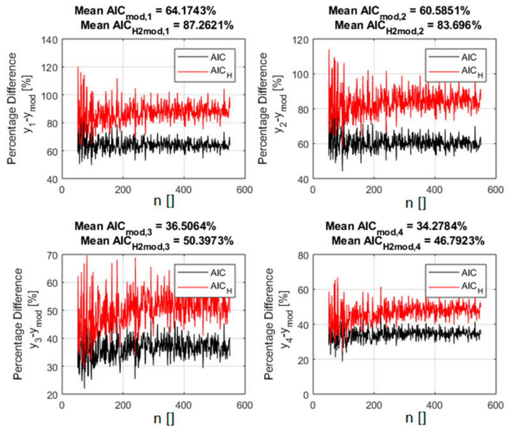

Gaussian Noise 10% - BIC (Figures S10–S12)

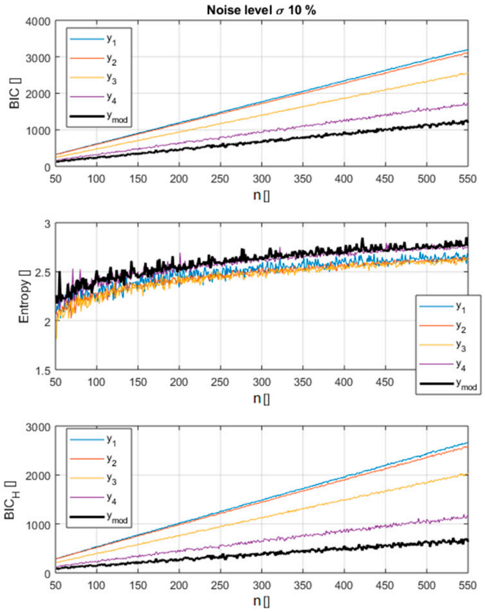

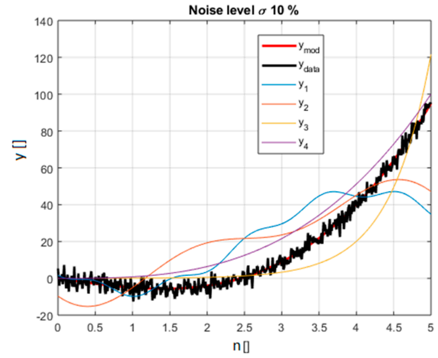

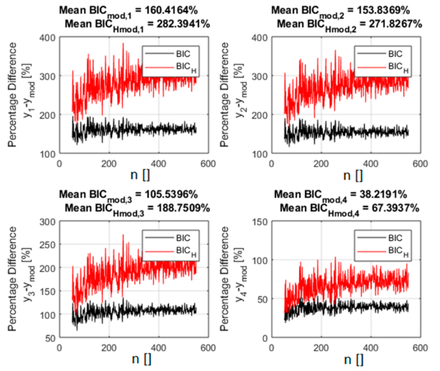

Gaussian Noise 20% - BIC (Figures S16–S18)

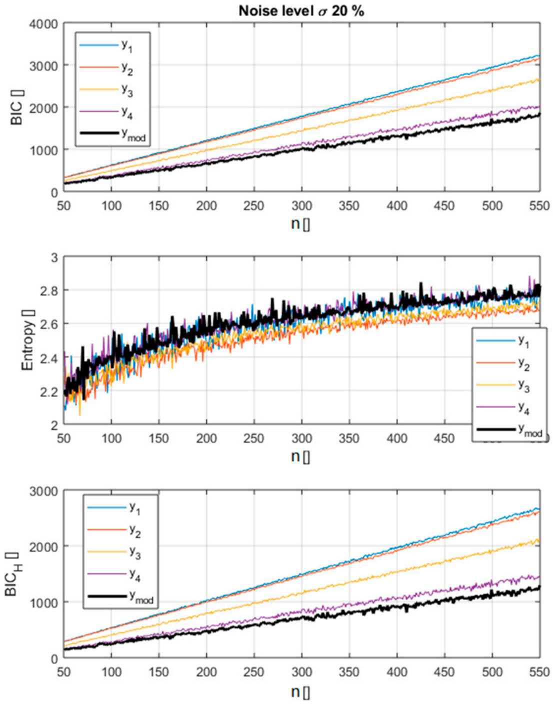

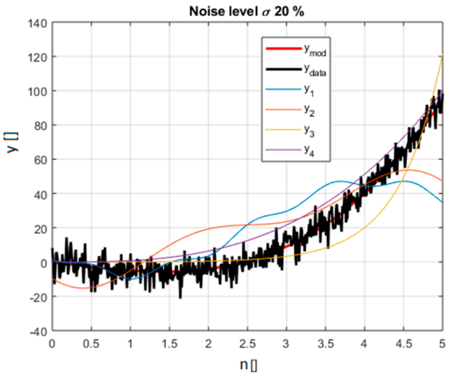

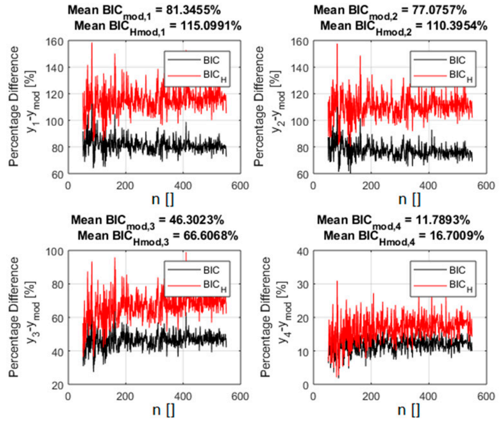

Gaussian Noise 25% - BIC (Figures S19–S21)

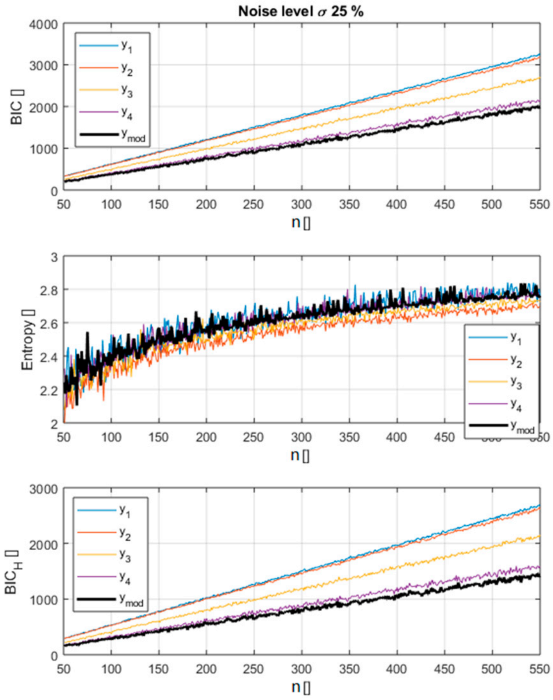

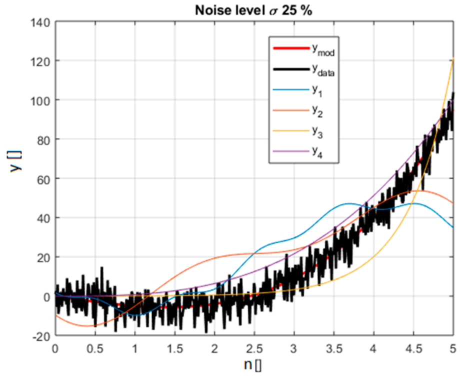

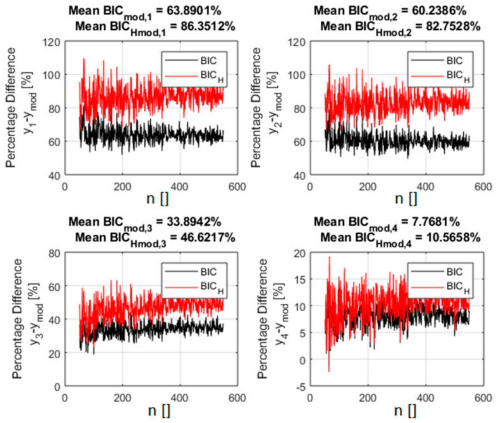

Appendix B: Scan in the noise for the models belonging to the class of Power Law monomials

Gaussian Noise 5% - AIC (Figures S22–S24)

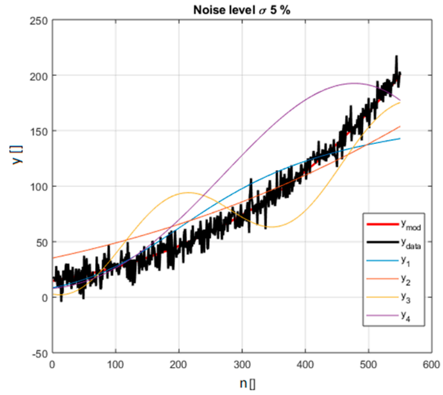

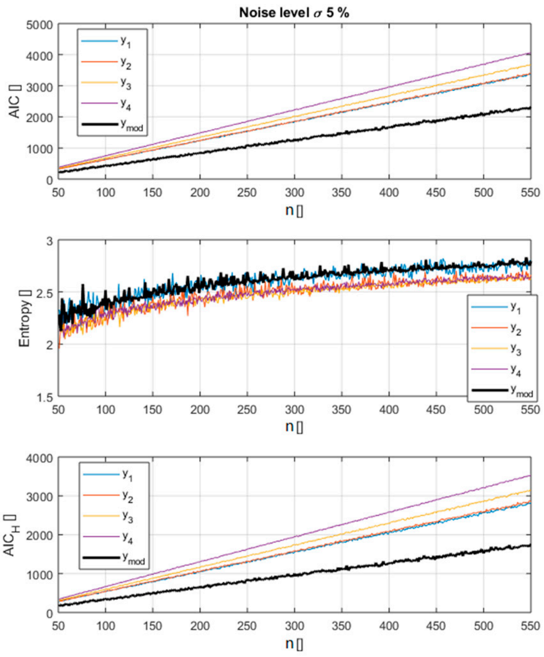

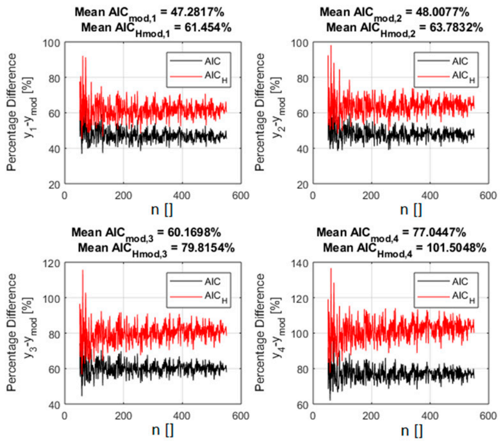

Gaussian Noise 10% - AIC (Figures S25–S27)

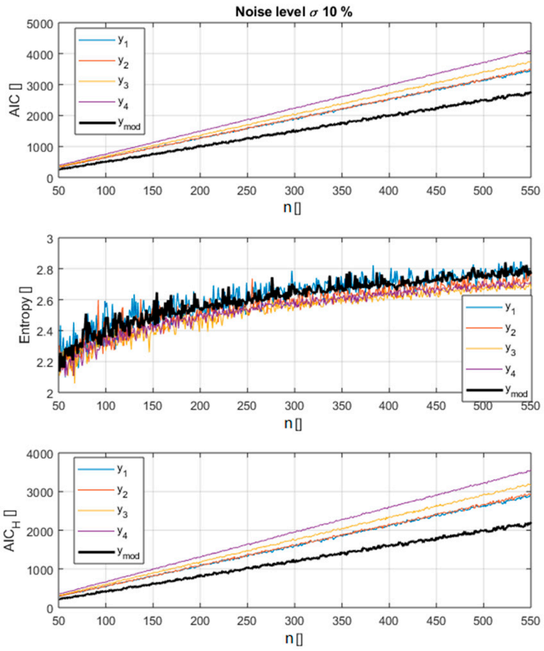

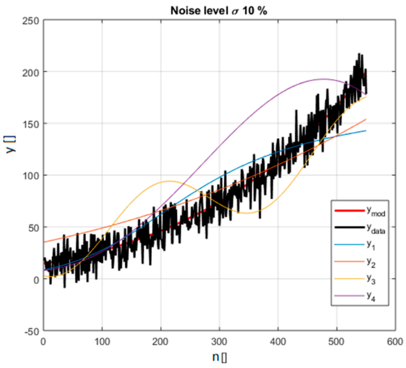

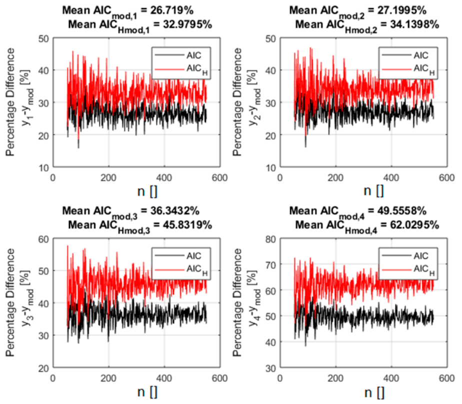

Gaussian Noise 20% - AIC (Figures S28–S30)

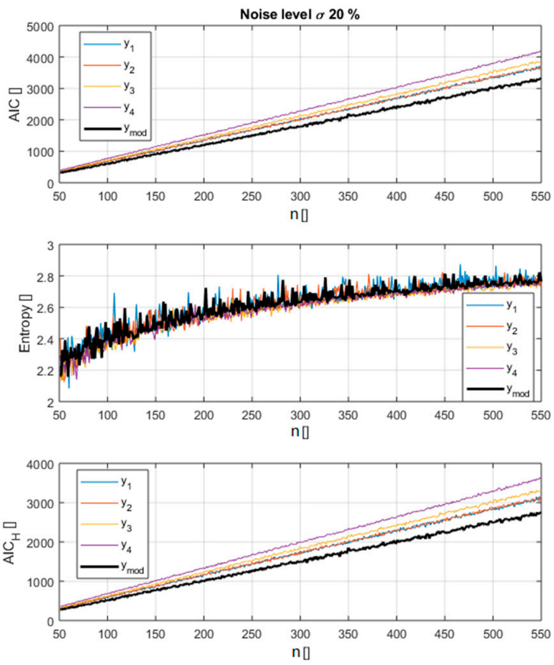

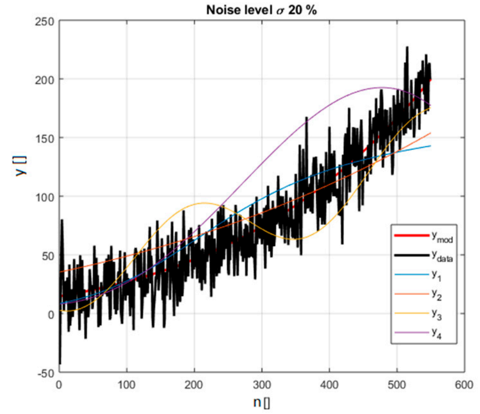

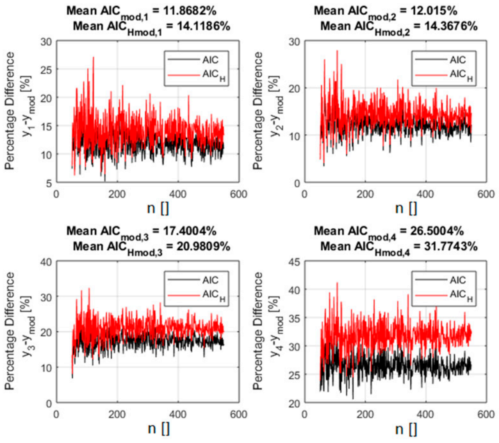

Gaussian Noise 25% - AIC (Figures S31–S33)

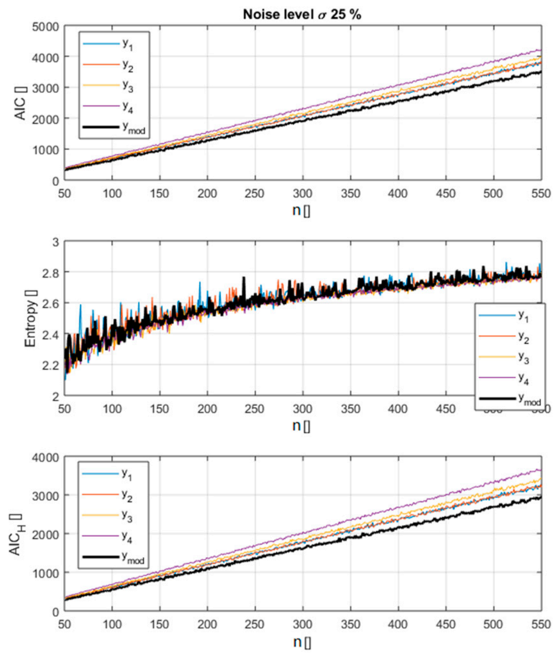

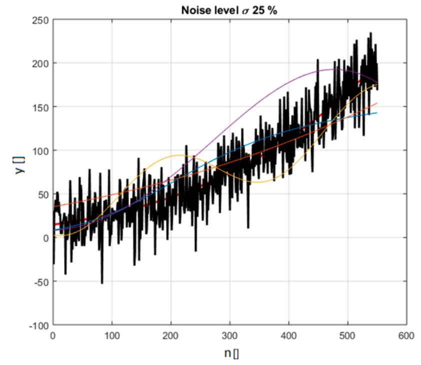

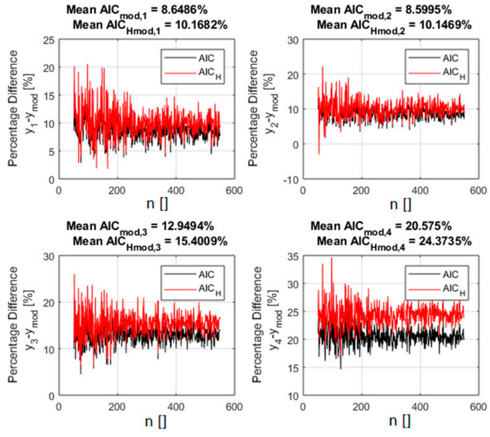

Gaussian Noise 5% - BIC (Figures S34–B36)

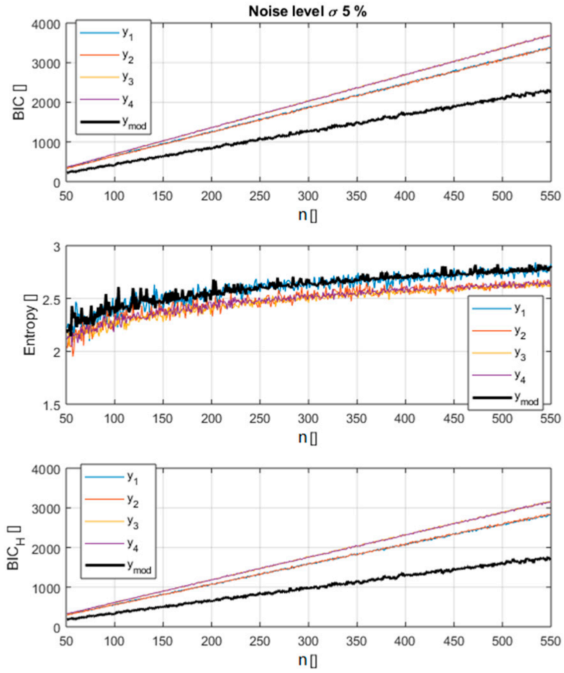

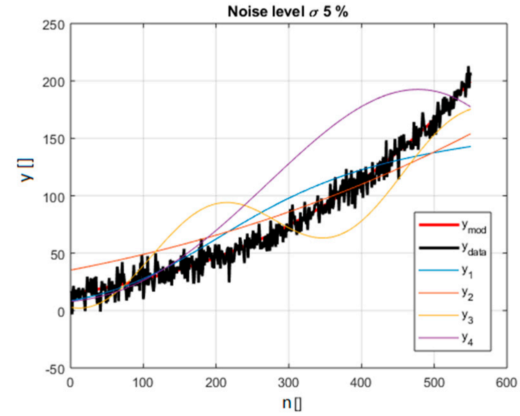

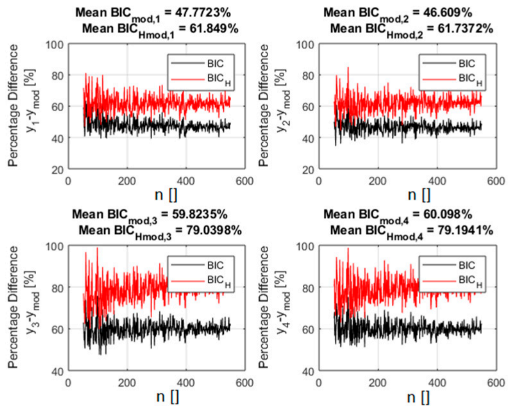

Gaussian Noise 10% - BIC (Figures S37–S39)

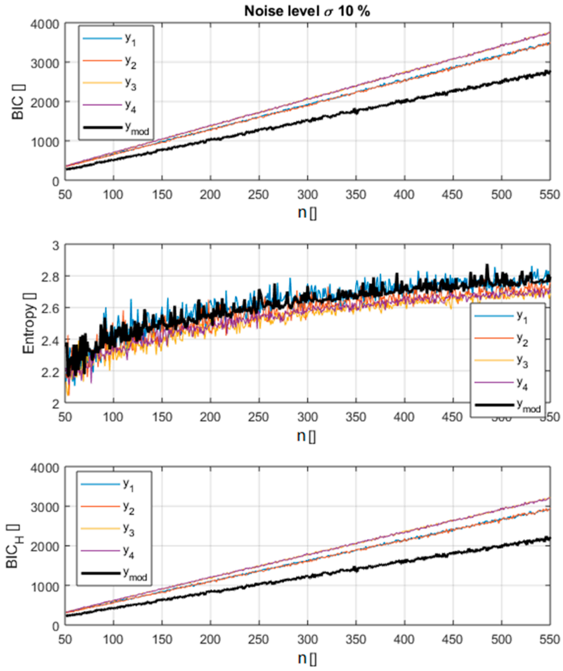

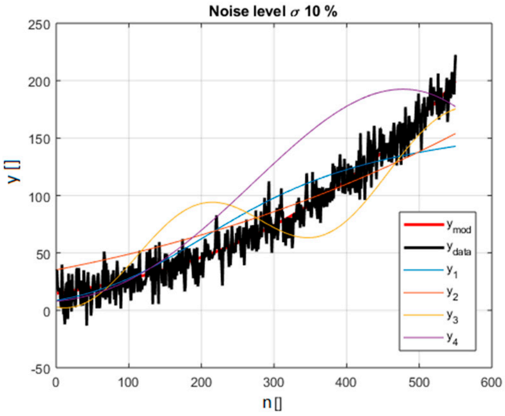

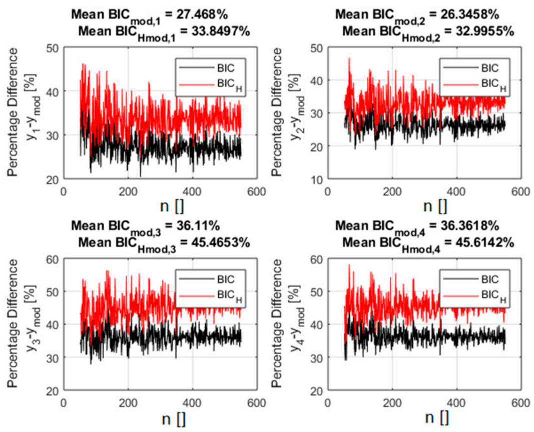

Gaussian Noise 20% - BIC (Figures S42–S45)

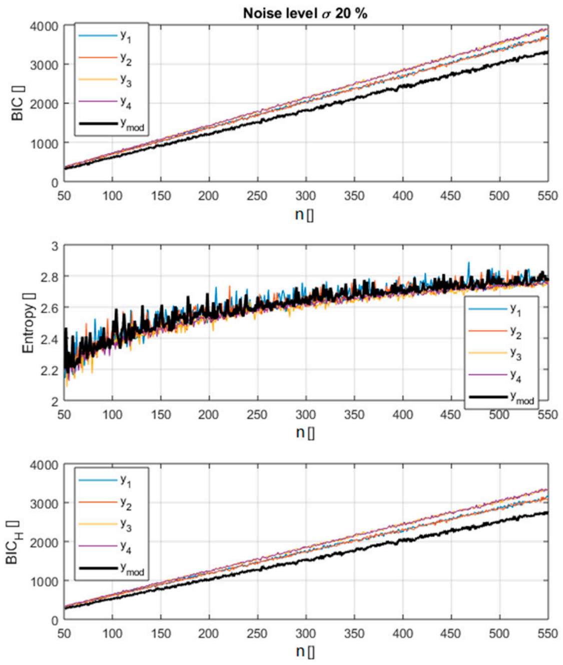

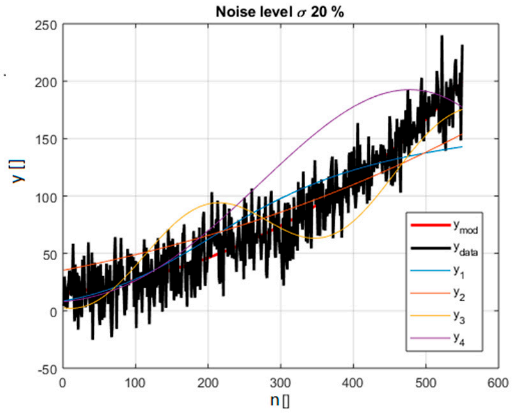

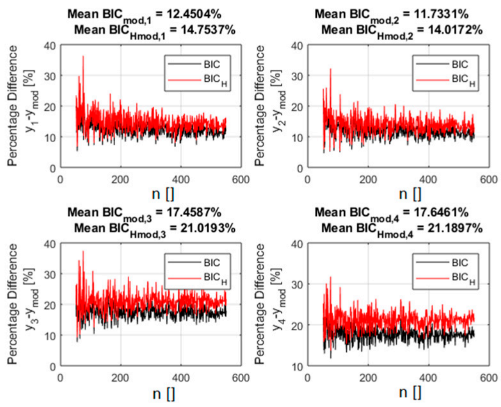

Gaussian Noise 25% - BIC (Figures S46–S48)

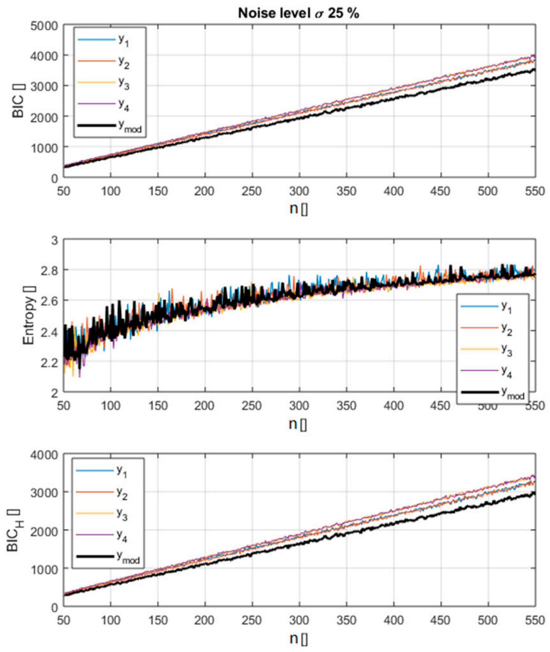

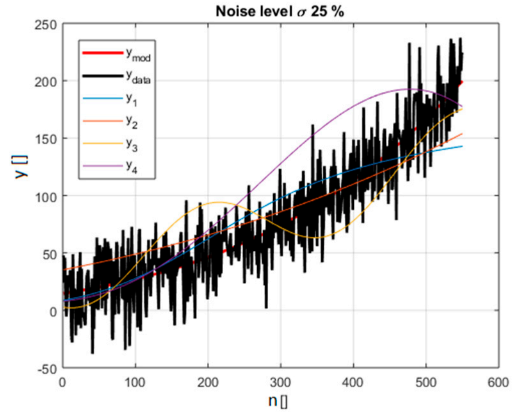

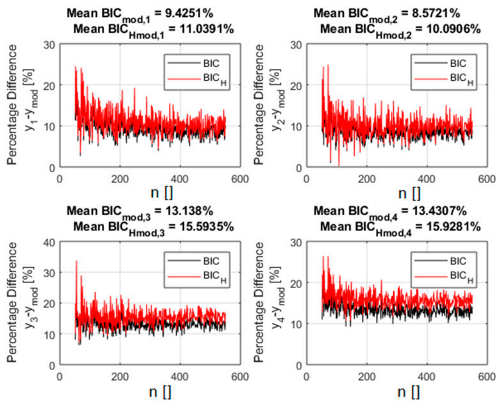

Supplement: Supplementary file 1 [file entropy-21-00394-s001.pdf]
